# Workload Differentiates Breast Surgical Procedures: NSM Associated with Higher Workload Demand than SSM

**DOI:** 10.1245/s10434-019-08159-0

**Published:** 2020-01-08

**Authors:** M. Susan Hallbeck, Katherine E. Law, Bethany R. Lowndes, Anna R. Linden, Melissa Morrow, Renaldo C. Blocker, Stephen M. Cain, Amy C. Degnim, Tina J. Hieken, James W. Jakub, Jennifer M. Racz, David R. Farley, Heidi Nelson, Judy C. Boughey

**Affiliations:** 1 Department of Health Sciences Research, Mayo Clinic, Rochester, MN USA; 2 Robert D. and Patricia E. Kern Center for the Science of Health Care Delivery, Mayo Clinic, Rochester, MN USA; 3 Department of Surgery, Mayo Clinic, Rochester, MN USA; 4 Department of Neurological Sciences, University of Nebraska Medical Center, Omaha, NE USA; 5 Department of Mechanical Engineering, University of Michigan, Ann Arbor, MI USA

## Abstract

**Background:**

Breast surgery has evolved with more focus on improving cosmetic outcomes, which requires increased operative time and technical complexity. Implications of these technical advances in surgery for the surgeon are unclear, but they may increase intraoperative demands, both mentally and physically. We prospectively evaluated mental and physical demand across breast surgery procedures, and compared surgeon ergonomic risk between nipple-sparing (NSM) and skin-sparing mastectomy (SSM) using subjective and objective measures.

**Methods:**

From May 2017 to July 2017, breast surgeons completed modified NASA-Task Load Index (TLX) workload surveys after cases. From January 2018 to July 2018, surgeons completed workload surveys and wore inertial measurement units to evaluate their postures during NSM and SSM cases. Mean angles of surgical postures, ergonomic risk, survey items, and patient factors were analyzed.

**Results:**

Procedural duration was moderately related to surgeon frustration, mental and physical demand, and fatigue (*p* < 0.001). NSMs were rated 23% more physically demanding (*M* = 13.3, SD = 4.3) and demanded 28% more effort (*M* = 14.4, SD = 4.6) than SSMs (*M* = 10.8, SD = 4.7; *M* = 11.8, SD = 5.0). Incision type was a contributing factor in workload and procedural difficulty. Left arm mean angle was significantly greater for NSM (*M* = 30.1 degrees, SD = 6.6) than SSMs (*M* = 18.2 degrees, SD = 4.3). A higher musculoskeletal disorder risk score for the trunk was significantly associated with higher surgeon physical workload (*p* = 0.02).

**Conclusion:**

Nipple-sparing mastectomy required the highest surgeon-reported workload of all breast procedures, including physical demand and effort. Objective measures identified the surgeons’ left upper arm as being at the greatest risk for a work-related musculoskeletal disorder, specifically from performing NSMs.

## Background

Breast surgery has evolved over the years—from radical mastectomy to modified radical mastectomy to breast conserving surgery; however, many women still require or elect to pursue mastectomy. Mastectomy options have also changed from total mastectomy to skin-sparing mastectomy and, most recently, to increasing use of nipple-sparing mastectomy. These advances improve the cosmetic outcome for patients, yet require additional operative time and increased technical complexity to preserve greater amounts of the breast envelope, potentially through hidden incisions. As a result, such technical advancements in surgery can affect surgeons’ mental and physical demand—and thus workload—during the procedure. The implications of these advances on the surgeon, however, are unclear.

Limited research on workload in surgery exists. Workload, a construct developed to explain the human ‘cost’ to perform a task, takes into consideration the mental and physical requirements necessary to accomplish a task.^1^ The NASA-Task Load Index (NASA-TLX)^1^ is a validated tool that quantifies an individual’s perception of their workload across multiple domains.^2^ NASA-TLX and similar instruments have been utilized to understand the implications of laparoscopic^3^ and robotic operations^4^,^5^ on surgeon workload as well as to study specific surgical specialties, such as bariatric^6^ and colorectal surgery.^7^ Most recently, our work documented varying workload across ten surgical specialties.^8^

Surveys and systematic reviews of surveys note that up to 95% of surgeons experience work-related pain associated with poor ergonomics during procedures. This pain experienced by surgeons, most of whom are minimally invasive specialists, may lead to their premature retirement and reduce the availability and accessibility of specialized procedures to patients.^9^^–^12 A recent meta-analysis observed that musculoskeletal injuries were the most common reason for absenteeism among surgeons.^13^ Several studies have also found approximately 50% of surgeons fear they will have a shortened career due to musculoskeletal disorders, with a significant correlation between this fear of a shortened career and burnout.^14^,^15^ Jackson and colleagues employed NASA-TLX and SURG-TLX questions to ask breast surgeons to compare nipple-sparing mastectomy and skin-sparing mastectomy, finding that surgeons reported greater physical symptoms, mental strain, and fatigue with nipple-sparing mastectomy than skin-sparing mastectomy.^16^ Together, these findings raise concern that discomfort along with increased cognitive and technical demand over the course of a breast surgeon’s career may lead to musculoskeletal disorders and burnout and ultimately shorten surgical careers.

Specialty-specific data are needed to evaluate the impact of different procedures as well as varying approaches to the same procedure. While most breast surgeons will comment that nipple-sparing mastectomy is technically more challenging and more strenuous on their bodies, objective data in this domain is lacking. The goal of the current study was to focus on breast surgery procedures and evaluate mental and physical demand across procedures, as well as compare the ergonomics for the surgeon between skin-sparing and nipple-sparing mastectomy using both subjective and objective measures.

## Methods

### Settings and Participants

A prospective observational study of breast surgeons at a large, quaternary care academic hospital was conducted under Institutional Review Board approval. Participation was optional and surgeons were instructed that they could opt out at any point during the study. Study results were anonymized so as to not identify individual surgeons.

This study was performed across two study phases. The first phase collected data between May 2017 and July 2017, where surgeons were asked to complete a modified NASA-Task Load Index (TLX) survey^1^ following each surgical case until they had completed 20 surveys to capture the self-reported mental and physical demand across breast operations including excisional biopsy, lumpectomy, total mastectomy, nipple-sparing mastectomies (NSM) and skin-sparing mastectomies (SSM). The second phase collected data between January 2018 and July 2018 and focused specifically on NSMs and SSMs. During this second phase, participants who perform both NSM and SSM procedures completed modified NASA-TLX surveys and were asked to wear a number of inertial measurement units (IMUs), wearable sensors that enable the measurement of motion, during two NSM cases and two SSM cases for a total of four surgical cases for each of the surgeons who performed both procedures.

### Phase 1: Self-Reported Survey Data

Surgeons were recruited and consented by email regarding the requirements of the study before completing an initial demographic questionnaire. Surveys were e-mailed to each surgeon on each surgical day with the date, case number of the day and operating room the case was performed in for all of their cases during May–July 2017. Survey development software (Qualtrics, Version 2017, Provo, UT) allowed for automated, secure delivery of workload questionnaires to the appropriate surgeon participant for each surgical day. The surgeons were asked to complete the survey as soon as possible after the case and ideally within 24 h of completion of the case.

### Modified NASA-TLX Workload Questionnaire

Workload was measured using the validated NASA-TLX,^1^ which was modified to include one question from the SURG-TLX,^17^ and one question on the difficulty of the procedure relative to the surgeon’s expectations.^8^ The original NASA-TLX contains six subscales (mental demand, physical demand, temporal demand, performance, frustration, and effort) that surgeons rate individually on a visual analog scale from 0 (very low) to 20 (very high). The added question from the SURG-TLX measured degree of distraction in the operative environment, on the same numeric scale (0–20). Expectation was rated according to whether the procedure was less difficult, as expected, or more difficult than expected.

### Patient-Related Factors

Patient and procedural data corresponding to the completed surveys were collected from the medical record. Data for patients that declined use of their medical data for research purposes were not included. Patient and procedural data collected included: body mass index (BMI), age, gender, American Society of Anesthesiologists (ASA) category, procedure type, and procedural duration (i.e., incision to closure duration).

### Phase 2: Self-Reported Survey Data and Postural Data for Ergonomic Risk Scores for NSM and SSM

During the second data collection phase, NSM and SSM procedures were targeted for collection. The survey questionnaire remained the same from Phase 1 of data collection, with the inclusion of additional questions. Two added questions included the degree of difficulty of the procedure,^18^ as well as one question on surgeon fatigue level,^19^ both rated from 0 (very low) to 20 (very high). Additional questions about procedural details, including type of incision and breast size, were included. Four of the six breast surgeons from Phase 1 routinely perform both NSM and SSM procedures and volunteered to wear IMUs during two cases each of NSM and SSM procedures to evaluate their postures during the procedures.^20^ Patient and procedural data were acquired using the same methodology from Phase 1.

### Inertial Measurement Unit Wearable Sensors

Before the surgical procedure, a researcher affixed the IMU wearable sensors on the surgeon and guided the surgeon through a series of functional calibration movements and postures similar to methods used previously.^21^^–^23 The IMU (APDM, Inc., Portland, OR) is a small (4.8 × 3.7 × 1.4 cm) electronic device that measures acceleration, angular velocity and magnetic field at 128 Hz, enabling estimates of body segment orientations and body postures.^24^ Four sensors were placed on the surgeon: one each on the right upper arm, left upper arm, posterior head and posterior trunk. While the surgeon performed the NSM or SSM procedure, IMU data were collected and later processed and analyzed using a custom algorithm developed in MATLAB (Mathworks, Natick, MA). At the end of the procedure, the IMUs were removed from surgeon participants who were also asked to complete the Phase 2 subjective workload survey within 24 h via a secure link from their email.

### IMU Data Analysis

During post-processing, the custom analysis algorithm first used the IMU data collected during the functional calibration movements and postures to define the orientations of the body segments (right upper arm, left upper arm, trunk and head) relative to the IMUs, ensuring that effects of sensor placement were minimized.^21^^–^23 Next, the IMU data was used to estimate the orientation of each body segment throughout the procedure.^24^,^25^ From the orientations of each body segment, the segment deviation angles or angles of the segment superior-inferior axes relative to gravity were calculated. Segment deviation angles were stratified into ergonomic risk categories using a modified Rapid Upper Limb Assessment (RULA) protocol. RULA involves four levels, with level 1 representing the lowest risk of injury and level 4 representing the highest.^26^ The percentage of time spent in each RULA level during the procedure and an overall risk score by body part (neck, upper arm/shoulder and trunk) were calculated. The overall risk score was a time-weighted average of percentage of time spent in each RULA level (1 = lowest to 4 = maximum).

### Statistical Analysis

Statistical analyses were performed using IBM SPSS Statistics (Version 22, Armonk, NY) and Microsoft Excel (Seattle, WA). Descriptive statistics performed included means (*M*) with standard deviations (SD) and medians (Mdn) with interquartile ranges (IQR). Procedures were categorized as NSM, SSM, total mastectomy, lumpectomy, and excisional biopsy. Analyses of variance (ANOVA) examined differences in workload between procedure types. Correlations were used to evaluate relationships between workload questionnaire items and patient factors as well as questionnaire items and IMU output. *p* values < 0.05 were considered significant for all analyses.

## Results

### Phase 1: Survey of Surgical Breast Procedures

Six surgeons (4 female, 6 right-handed) participated in Phase 1 of the study and completed 98 surveys. A majority of surveys (75/98, 77.5%) were completed within 24 h of the case. Most participants (66.8%) were considered very experienced surgeons, with participants reporting a median 18 years of surgical experience post residency. Patients were predominantly middle-aged and were categorized as ASA Class II or III (Table 1). Both patient breast size and BMI were significantly different across procedure types (*p* < 0.001; *p* = 0.02, respectively). Patients who underwent SSMs had significantly larger breast sizes than other procedure types, with 17.4% of SSM patients falling in the largest breast size category, compared to only 2.3% of NSM patients (*p* < 0.001). Additionally, SSM patients had higher BMIs (*M* = 29.3, SD = 6.1, *p* = 0.02) than other procedure types.

**Table 1:** Patient demographics by procedure type

Characteristic (median, [Q1, Q2])	Nipple sparing (NSM) *n* = 43	Skin sparing (SSM) *n* = 46	Total mastectomy *n* = 27	Lumpectomy *n* = 40	Excisional biopsy *n* = 16	Overall *n* = 172
Age (years)	50.2 (43.4, 58.9)	48.5 (40.3, 60.1)	60 (51.2, 74.5)	61.2 (52.6, 69.6)	53.5 (51.4, 60.2)	54.4 (46.3, 64.0)
BMI	25.3 (21.9, 28.3)	27.7 (24.4, 34.1)	28.2 (23.8, 36.3)	29.7 (23.8, 35.6)	25.8 (22.9, 31.4)	27.4 (23.7, 32.2)
Height (cm)	163 (157.3, 168)	167.3 (163.6, 169.5)	161 (156.5, 167.3)	160 (156, 166)	161 (160, 164)	163 (158, 168)
Weight (kg)	65.8 (61.1, 79.7)	78.8 (67.6, 96.3)	72.9 (61.7, 93.8)	72.4 (63, 90.9)	67 (62.9, 81)	72 (62.1, 87.0)
Surgical duration (min)	242.3 (68.7)	255.4 (64)	191.9 (50.3)	120.2 (36)	76.5 (25.5)	199.8 (84)
Breast size (*n* [%])						
Small (A)	5 (11.6%)	2 (4.3%)	5 (18.5%)	–	1 (6.3%)	13 (7%)
Medium (B/C)	21 (48.9%)	7 (15.2%)	2 (7.4%)	3 (7.5%)	1 (6.3%)	34 (19.8%)
Large (D-DD)	7 (16.3%)	16 (34.8%)	2 (7.4%)	2 (5%)	1 (6.3%)	28 (16.3%)
Extra large (> DD)	1 (2.3%)	8 (17.4%)	7 (25.9%)	2 (5%)	–	18 (10.5%)
Missing	9 (20.9%)	13 (28.3%)	11 (40.7%)	33 (82.5%)	13 (81.1%)	79 (45.9%)
ASA category (*n* [%])						
I	4 (9.3%)	1 (2.2%)	1 (3.7%)	2 (5%)	1 (6.3%)	9 (5.2%)
II	32 (74.4%)	32 (69.6%)	13 (48.1%)	22 (%)	12 (75%)	111 (64.5%)
III	7 (16.3%)	10 (21.7%)	12 (44.4%)	15 (37.5%)	3 (18.7%)	47 (27.3%)
IV	–	–	1 (3.7%)	1 (2.5%)	–	2 (1.4%)
Missing	0 (%)	3 (6.5%)	0 (%)	0 (%)	0 (%)	3 (1.7%)

Surgical duration differed significantly across procedures. NSMs and SSMs were both significantly longer than all other procedure types [*F*(4,144) = 45.9, *p* < 0.001; Table 2]. Procedural duration was moderately related to surgeon frustration (*r* = 0.3, *p* < 0.001), mental and physical demand (*r* = 0.29, *p* < 0.001; *r* = 0.48, *p* < 0.001, respectively), as well as fatigue (*r* = 0.3, *p* < 0.001). Across procedure types, there was a significant difference in self-reported degree of difficulty (*p* = 0.02), with NSM rated the highest (*M* = 12.4, SD = 4.5, *p* = 0.02). The average patient age for procedures rated “more difficult than expected” (*M* = 51 years old, SD = 12.2) was significantly lower than the average patient age for procedures rated “as difficult as expected” (*M* = 59 years old, SD = 12.6, *p* = 0.003). While age also varied significantly by procedure type, with younger patients undergoing NSMs (*M* = 51 years old, SD = 11.3) and older patients undergoing lumpectomies (*M* = 62 years old, SD = 11.4), a two-way ANOVA identified no significant interaction between procedure type and expectation of difficulty (*p* > 0.1).

**Table 2:** Workload by procedure type for phase one and two

Workload domain (mean [SD])	Nipple sparing (NSM) *n* = 43	Skin sparing (SSM) *n* = 46	Total mastectomy *n* = 27	Lumpectomy *n* = 40	Excisional biopsy *n* = 16	Overall *n* = 172
Surgical duration (min)	242.3 (68.7)	255.4 (64)	191.9 (50.3)	120.2 (36)	76.5 (25.5)	199.8 (84)
Overall workload (max = 100)	55.7 (15.3)	48.4 (17.1)	37.0 (16.3)	33.5 (13.7)	22.7 (14)	42.6 (18.7)
Mental demand (max = 20)	11.1 (4.4)	10.2 (5.2)	8.6 (4.1)	8.8 (4.4)	6.5 (4.9)	9.5 (4.7)
Physical demand (max = 20)	13.3 (4.3)	10.8 (4.7)	7.9 (4.3)	7.2 (3.4)	5.3 (3.4)	9.6 (4.9)
Temporal demand (max = 20)	7.34 (4.5)	6.11 (5)	6.15 (3.9)	5.28 (4.6)	3.5 (4.2)	6.0 (4.6)
Effort (max = 20)	14.4 (4.6)	11.8 (5)	9.2 (4.5)	9.5 (4.2)	6.5 (4.1)	11.0 (5.1)
Performance (max = 20)	2.8 (2.3)	3.5 (3.4)	3.0 (3.7)	2.7 (4.2)	1.29 (0.8)	2.9 (3.3)
Frustration (max = 20)	7.7 (4.9)	7.6 (6)	5.8 (5)	4.9 (4.9)	3.44 (3.5)	6.3 (5.3)
Distraction (max = 20)	7.2 (5.3)	5.8 (4.1)	4.0 (3.2)	3.6 (3.8)	2.1 (2.9)	5.0 (4.4)

### Phase 2

Five surgeons (4 female, 5 right-handed) completed additional surveys (*n* = 74, 94.6% completion within 24 h) solely on NSM and SSM procedures. IMU data were collected from four of the five surgeons intraoperatively for objective physical posture data for NSM and SSM procedures. For the cases collected during Phase 2, patient BMI was again significantly different between procedure types [*F*(1,82) = 8.7, *p* = 0.004], with a higher mean BMI for SSMs (29.3, SD = 6.1) than NSMs (25.8, SD = 4.6).

**Fig. 1:**
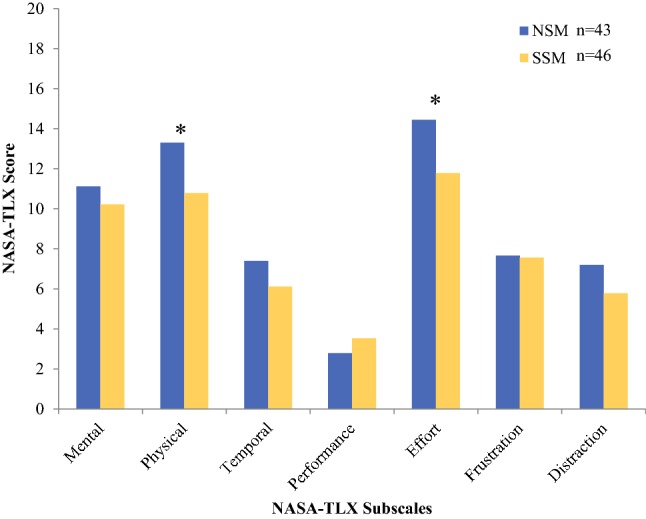
Average NASA-TLX scores according to NSM and SSM procedures. *p < 0.05 or statistically significantly different NASA-TLX scores by procedure (NSM vs. SSM) for the subscales for physical demand and perceived effort

Across both Phase 1 and Phase 2, surgeon-reported workload across the various NASA-TLX subscales differed by procedure (Table 3). NSM procedures were rated 23% more physically demanding (*M* = 13.3, SD = 4.3) than SSM procedures (*M* = 10.8, SD = 4.7, *p* = 0.01). In addition, surgeons reported NSMs required significantly more (28% higher) effort (*M* = 14.4, SD = 4.6) than SSMs (*M* = 11.8, SD = 5.0, *p* = 0.01). Degree of difficulty was also 18% higher for NSMs (*M* = 12.4, SD = 4.5) than SSMs (*M* = 10.5, SD = 3.9), trending towards significance (*p* = 0.07; Fig. 1).

**Table 3:** Posture differences and risks by procedure type

	NSM	SSM	*p* value
Left upper arm mean angle*	30.1 (6.6)	18.2 (4.3)	0.01
Left upper arm risk score*	1.87 (0.24)	1.39 (0.18)	< 0.001
Right upper arm mean angle	26.2 (8.3)	23.4 (9.6)	0.848
Right upper arm risk score	1.7 (0.36)	1.59 (0.4)	0.874
Neck mean angle	27.3 (7.5)	32.9 (10.4)	0.735
Neck risk score^#^	2.54 (0.31)	2.78 (0.25)	0.080
Trunk mean angle	15.2 (4.2)	17.7 (7.3)	0.408
Trunk risk score	1.93 (0.32)	2.10 (0.43)	0.775

^#^Risk score on scale of 1–4. A score ≥ 2.5 is considered risky

*p < 0.01

**Fig. 2:**
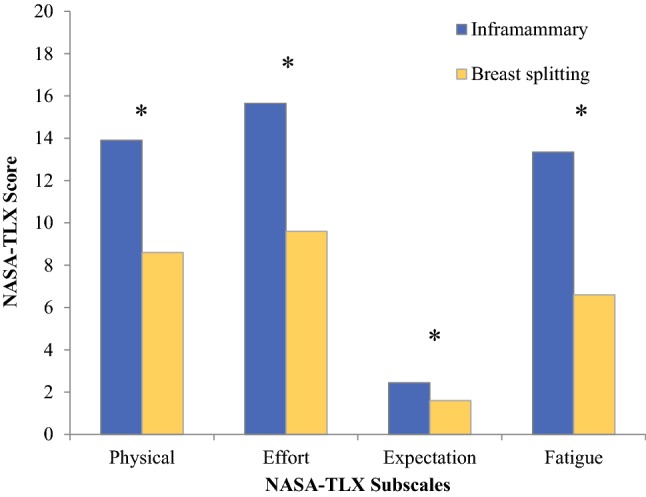
Average NASA-TLX scores by incision type for NSM procedures. *p < 0.05 or statistically significant difference between inframammary and breast-splitting approaches for NSM for the 4 NASA-TLX subscales

Further analysis of the data indicated the incision location proved to be a contributing factor in workload and expected difficulty. NSMs employing an inframammary incision required significantly higher physical demand (*M*_inframammary_ = 13.9, SD = 4.1; *M*_breast-splitting_ = 8.6, SD = 4.2, *p* = 0.02), effort (*M*_inframammary_ = 15.7, SD = 3.8; *M*_breast-splitting_ = 5.8, SD = 3.8, *p* = 0.01) and fatigue (*M*_inframammary_ = 13.4, SD = 4.8; *M*_breast-splitting_ = 6.6, SD = 3.3, *p* = 0.02) compared to breast splitting incisions (Fig. 2). Additionally, NSM via inframammary fold incision were more often rated “more difficult than expected” (43%) than NSMs that used a breast splitting incision (20%; *p* = 0.001). SSMs employing other incision types (e.g., peri-areolar incision) required significantly higher temporal demand than either ellipse around areola (*M*_other_ = 12.3, SD = 0.58; *M*_ellipse_ = 3.4, SD = 2, *p* = 0.001) or reduction (wise) pattern incisions (*M*_reduction_ = 4.4, SD = 3.9, *p* < 0.001). No significant differences were observed in the case distribution of incision type for SSM by expected difficulty (*p* > 0.05).

IMU data identified a high risk for neck musculoskeletal disorders with both NSM and SSM, with risk scores exceeding 2.5 (Table 3). This threshold was determined by previous occupational ergonomic research that linked musculoskeletal pain and disorders with exposure to neck and arm postures considered to be in high risk categories.^27^ In addition, several postural differences were observed between NSM and SSM procedures (Table 3). Surgeons demonstrated a significantly higher angle of the left arm while performing an NSM (*M* = 30.1°, SD = 6.6) than during the SSMs (*M* = 18.2°, SD = 4.3). Additionally, a higher musculoskeletal disorder risk score for the chest was significantly associated with higher surgeon physical workload (*r* = 0.57, *p* = 0.02). The association between mean angle of the left arm and self-reported physical workload, as well as between left arm and self-reported performance, also trended towards significance (*r* = 0.48, *p* = 0.06; *r* = 0.50, *p* = 0.06).

## Discussion

This study of 6 surgeons across 172 breast procedures demonstrates that NSM had the highest overall workload of all breast procedures and required the highest physical demand and effort from the surgeon. Additionally, NSMs performed through an inframammary incision were more than one and a half times more physically demanding, elicited more than twice the fatigue levels and required more than 2 and a half times more effort than using a breast splitting incision. Through IMU assessment of surgeon positioning during NSMs, the surgeon’s left upper arm as well as shoulder were at the highest ergonomic musculoskeletal disorder risk. Within our measurement of risk for the upper arms, a high risk rating is reflective of sustained upper arm postures of high elevation. Exposure of the shoulder joint to persistent high elevation is associated with soft tissue pathology such as tendinopathy and tendon tears of shoulder musculature of the rotator cuff.^28^,^29^

While NSM is likely here to stay and demand for NSM will continue to rise as patients seek out cosmetic-oriented mastectomy procedures, the higher workload associated with this procedure identifies a potential source for surgeon burnout, both physically and mentally. Thus, the maintenance and preservation of the work force of breast surgeons is critical. Understanding the workload of NSM should be incorporated when a procedure-specific CPT (Current Procedural Terminology) code is developed, as currently mastectomy procedures—total mastectomy, skin-sparing mastectomy, or nipple-sparing mastectomy—are all billed under the same procedure code. It is also important for hospitals and practices to be aware of the potential for surgeon physical burnout related to musculoskeletal disorders from recurring poor ergonomics^16^ as well as mental burnout from the cognitive workload of increasingly complex procedures. For an individual surgeon, awareness of workload demands can also inform scheduling decisions and allow each to determine the appropriate mix of operative cases per day. Because work-related injury in surgeons is an understated issue, these findings could be a potential source of objective data when submitting disability claims or initial justification for organizations such as Occupational Safety and Health Administration (OSHA) to protect the surgical profession.

The inframammary fold incisions used in NSMs yielded significantly higher physical demand, effort, and fatigue from surgeons. This is likely related to poorer visualization of the operative field and working from the inferior aspect of the patient’s breast—up the breast mound towards the nipple and then back down the breast mound on the other side to the clavicle—requiring changes in angle with poorer exposure and a long total flap length. While a breast splitting incision allows the surgeon to approach the breast from the anterior aspect in the middle of the breast mound—more similar to a skin-sparing mastectomy—and dissect shorter skin flaps down the breast mound from the top, it still produces considerably lower workload demands.

This study identified the surgeons’ left upper arm as being at the greatest risk for work-related musculoskeletal disorder—specifically, while performing NSMs. For right-handed surgeons, this is likely related to using the left hand for anterior traction on the breast and skin flaps for exposure, and identifies a need for development of better ways to provide this traction for this procedure. Prolonged shoulder abduction with elevation of the arm above 45° (i.e., RULA level 2) can pose moderate risk, while abduction with elevation greater than 90° (i.e.,. RULA level 4) can pose extreme risk. According to the RULA risk ratings, a level 3 risk indicates that the task should be changed soon, while a level 4 risk indicates that change should be implemented immediately.^30^ Understanding the musculoskeletal strain on the body can help guide interventions to minimize long-term repetitive strain injury. Use of microbreaks^28^,^29^ may be beneficial, as may a change in technical approach.^31^,^32^ Microbreaks encourage the surgeon and the surgical team to change position and stretch at designated periods throughout a procedure and they have been shown to increase physical function and mental focus.^33^ Our data indicate that these interventions may not be necessary during lumpectomy or excisional biopsy procedures, but more appropriate for skin-sparing and nipple-sparing mastectomies—the longer and more fatiguing procedures. The poor neck position seen during both NSM and SSM procedures can also be exacerbated with the additional weight of a head light. In the second phase of this study, 2 of the 4 surgeons routinely wore a head light for visualization of the operative field. While often not considered heavy, any additional weight on the head transfers to the fulcrum point—the neck—which exacerbates the impact of poor neck positioning in terms of muscle and joint strain, thus contributing to higher risk for musculoskeletal disorders.^34^

While the impact of incision type on workload was evaluated, further work is needed to determine whether different technical approaches for NSM can decrease the physical demand on the surgeon with minimal effect on patient outcomes. Several approaches can be used for NSM. Of the four surgeons in the ergonomic evaluation of this study, one utilizes a blunt face lift scissor dissection technique of the mastectomy planes in NSM; however, the sample size was too small to study the differences due to technique. Robotic NSM is currently emerging and it is not known what the impact of robotic NSM will be on operative workload. In colorectal surgery, the robotic approach has been shown to have a workload significantly less than laparoscopic colorectal surgery,^7^ and despite longer operative times, the robotic approach is more in line with the workload of an open approach.

This study builds upon the work performed by Jackson, et al. who published the first breast surgery ergonomics survey, especially with respect to mastectomy.^14^ While the Jackson study focused on subjective measures of surgeon pain and fatigue, the current study evaluates subjective measures but also provides objective measures on ergonomic strain that can lead to musculoskeletal disorders. Results from the current study echo Jackson et al. findings, specifically that physical demand while performing NSMs is significantly greater than that of SSMs. However, the current study also found that the inframammary approach may be a driver for that difference and further, that the objective ergonomic risk for MSDs is moderately high for the neck angles. Findings from both the Jackson study and this current study indicate that as patient demand for NSMs increases, the surgeons will also experience a greater workload. It is important to note that patient-driven demand for better cosmesis is not unique to breast surgery. For example, the single port access for laparoscopic cholecystectomies through the umbilicus yields better cosmesis.^35^,^36^ In a recent study analyzing workload for single incision (SILC) and multiple port laparoscopic cholecystectomies, the surgeon and main assistant that holds the endoscope experienced significantly higher workload, stress, and awkward body postures for the single port approach as compared to conventional laparoscopy.^37^ While procedures may produce better cosmetic outcomes, patient-driven complex procedures—such as SILC, NSM, and SSM—may also increase the overall workload of the surgeon and surgical team.

There are limitations to the current study. This research was performed in an academic, quaternary care facility with a high volume of complex procedures. A small sample size was used to assess both subjective and objective ergonomic measures. For generalizability, a larger sample size of surgeons across a longer time period is necessary. While subjective metrics were employed and participants were not blinded to the purpose of this study, this study also included IMUs to supplement objective measurement of surgeon posture. For the second phase of the study, a small sample size of surgeons and procedures was used. While procedures were paired to the surgeon, the technique utilized in NSM and SSMs was not standardized. Despite these limitations, this study was the first to employ both subjective and objective measures of surgeon ergonomics in breast surgery as well as indicate the need for changes to surgeon posture during nipple-sparing mastectomies.

## Conclusions

Nipple-sparing mastectomy required the highest overall self-reported workload of all breast procedures, including the highest physical demand and effort from the surgeon. Objective measures identified the surgeons’ left upper arm as being at greatest risk for work-related musculoskeletal disorder, specifically from performing NSMs. Demand for NSM will continue to rise as patients seek out mastectomy procedures with better esthetic outcomes, making maintenance and preservation of the breast surgeon workforce critical. Interventions that address higher workload and postures assumed during this procedure are necessary to reduce risk of musculoskeletal injury.

## Abbreviations

ASA: American Society of Anesthesiologists
BMI: Body mass index
IMU: Inertial measurement unit
IQR: Interquartile range
*M*: Mean
Mdn: Median
MSD: Musculoskeletal disorder
NASA-TLX: NASA-Task Load Index
NSM: Nipple-sparing mastectomy
RULA: Rapid Upper Limb Assessment
SD: Standard deviation
SSM: Skin-sparing mastectomy
SURG-TLX: Surgery-Task Load Index

## References

[1] Hart SG, Staveland LE (1988). Development of NASA-TLX (Task Load Index): results of empirical and theoretical research. Adv Psychol.

[2] Hart SG. NASA-task load index (NASA-TLX): 20 years later. In: Proceedings of the human factors and ergonomics society annual meeting. Los Angeles, CA: Sage Publications; 2006. p. 904–8.

[3] Yurko YY, Scerbo MW, Prabhu AS, Acker CE, Stefanidis D (2010). Higher mental workload is associated with poorer laparoscopic performance as measured by the NASA-TLX tool. Simul Healthc.

[4] Dulan G, Rege RV, Hogg DC (2012). Proficiency-based training for robotic surgery: construct validity, workload, and expert levels for nine inanimate exercises. Surg Endosc.

[5] Stefanidis D, Wang F, Korndorffer JR, Dunne JB, Scott DJ (2010). Robotic assistance improves intracorporeal suturing performance and safety in the operating room while decreasing operator workload. Surg Endosc.

[6] Ruiz-Rabelo JF, Navarro-Rodriguez E, Di-Stasi LL (2015). Validation of the NASA-TLX score in ongoing assessment of mental workload during a laparoscopic learning curve in bariatric surgery. Obes Surg.

[7] Law KE, Lowndes BR, Kelley SR (2018). NASA-task load index differentiates surgical approach: opportunities for improvement in colon and rectal surgery. Ann Surg.

[8] Lowndes BR, Forsyth KL, Blocker RC (2018). NASA-TLX assessment of surgeon workload variation across specialties. Ann Surg.

[9] Alleblas CC, De Man AM, Van Den Haak L, Vierhout ME, Jansen FW, Nieboer TE (2017). Prevalence of musculoskeletal disorders among surgeons performing minimally invasive surgery: a systematic review. Ann Surg.

[10] Dalager T, Søgaard K, Bech KT, Mogensen O, Jensen PT (2017). Musculoskeletal pain among surgeons performing minimally invasive surgery: a systematic review. Surg Endosc.

[11] Plerhoples TA, Hernandez-Boussard T, Wren SM (2012). The aching surgeon: a survey of physical discomfort and symptoms following open, laparoscopic, and robotic surgery. J Robot Surg.

[12] Stucky C-CH, Cromwell KD, Voss RK (2018). Surgeon symptoms, strain, and selections: systematic review and meta-analysis of surgical ergonomics. Ann Med Surg..

[13] Epstein S, Sparer EH, Tran BN (2018). Prevalence of work-related musculoskeletal disorders among surgeons and interventionalists: a systematic review and meta-analysis. JAMA Surg..

[14] Wells AC, Kjellman M, Harper SJF, Forsman M, Hallbeck MS (2018). Operating hurts: a study of EAES surgeons. Surg Endosc..

[15] Davila V, Hallbeck M, Stone W, Money S (2019). Physical discomfort, professional satisfaction, and burnout in vascular surgeons. J Vasc Surg..

[16] Jackson R, Sanders T, Park A (2017). Prospective study comparing surgeons’ pain and fatigue associated with nipple-sparing vs skin-sparing mastectomy. Ann Surg Oncol..

[17] Wilson MR, Poolton JM, Malhotra N, Ngo K, Bright E, Masters RS (2011). Development and validation of a surgical workload measure: the surgery task load index (SURG-TLX). World J Surg.

[18] Vassiliou MC, Feldman LS, Andrew CG, Bergman S, Leffondré K (2005). A global assessment tool for evaluation of intraoperative laparoscopic skills. Am J Surg..

[19] Kuorinka I, Jonsson B, Kilbom A (1987). Standardised Nordic questionnaires for the analysis of musculoskeletal symptoms. Appl Ergon.

[20] Morrow MM, Lowndes B, Fortune E, Kaufman KR, Hallbeck MS (2017). Validation of inertial measurement units for upper body kinematics. J Appl Biomech..

[21] De Vries W, Veeger H, Cutti A, Baten C, Van Der Helm F (2010). Functionally interpretable local coordinate systems for the upper extremity using inertial & magnetic measurement systems. J Biomech..

[22] Ricci L, Formica D, Sparaci L (2014). A new calibration methodology for thorax and upper limbs motion capture in children using magneto and inertial sensors. Sensors.

[23] Cain SM, McGinnis RS, Davidson SP, Vitali RV, Perkins NC, McLean SG (2016). Quantifying performance and effects of load carriage during a challenging balancing task using an array of wireless inertial sensors. Gait Posture.

[24] Sabatini AM (2006). Quaternion-based extended Kalman filter for determining orientation by inertial and magnetic sensing. IEEE Trans Biomed Eng.

[25] Savage PG (1998). Strapdown inertial navigation integration algorithm design part 1: attitude algorithms. J Guid Control Dyn.

[26] McAtamney L, Corlett EN (1993). RULA: a survey method for the investigation of work-related upper limb disorders. Appl Ergon.

[27] Nordander C, Hansson G-Å, Ohlsson K (2016). Exposure–response relationships for work-related neck and shoulder musculoskeletal disorders—analyses of pooled uniform data sets. Appl Ergon.

[28] Svendsen S, Bonde J, Mathiassen SE, Stengaard-Pedersen K, Frich L (2004). Work related shoulder disorders: quantitative exposure–response relations with reference to arm posture. Occup Environ Med.

[29] Svendsen SW, Gelineck J, Mathiassen SE (2004). Work above shoulder level and degenerative alterations of the rotator cuff tendons: a magnetic resonance imaging study. Arthritis Rheum.

[30] Bernard BP, Putz-Anderson V, National Institute for Occupational Safety and Health. Musculoskeletal disorders and workplace factors; a critical review of epidemiologic evidence for work-related musculoskeletal disorders of the neck, upper extremity, and low back. 1997. CDC/DHHS (NIOSH) Publication No. 97–141.

[31] Dorion D, Darveau S (2013). Do micropauses prevent surgeon’s fatigue and loss of accuracy associated with prolonged surgery? An experimental prospective study. Ann Surg.

[32] Park AE, Zahiri HR, Hallbeck MS (2017). Intraoperative “micro breaks” with targeted stretching enhance surgeon physical function and mental focus: a multicenter cohort study. Ann Surg.

[33] Hallbeck MS, Lowndes BR, Bingener J (2017). The impact of intraoperative microbreaks with exercises on surgeons: a multi-center cohort study. Appl Ergon.

[34] Catanzarite T, Tan-Kim J, Whitcomb EL, Menefee S (2018). Ergonomics in surgery: a review. Female Pelvic Med Reconstr Surg.

[35] Lowndes BR, Abdelrahman AM, Thiels CA (2018). Surgical team workload comparison for 4-port and single-port laparoscopic cholecystectomy procedures. Appl Ergon..

[36] Abdelrahman AM, Bingener J, Yu D (2016). Impact of single-incision laparoscopic cholecystectomy (SILC) versus conventional laparoscopic cholecystectomy (CLC) procedures on surgeon stress and workload: a randomized controlled trial. Surg Endosc.

[37] Abdelrahman AM, Lowndes B, Rand C (2017). Impact of robotic surgery versus laparoscopic surgery on surgeon musculoskeletal symptoms and workload: a systematic review and meta-analysis. Surg Endosc Other Interv Tech.

